# Predictors of intimate partner problem-related suicides among suicide decedents in Kentucky

**DOI:** 10.5249/jivr.v8i2.776

**Published:** 2016-07

**Authors:** Ashley L. Comiford, Wayne T. Sanderson, Lorie Chesnut, Sabrina Brown

**Affiliations:** ^*a*^University of Kentucky, College of Public Health, Kentucky, US.

## Abstract

**Background:**

Suicide is the 10th leading cause of death in the United States. Furthermore, intimate partner problems are amid the top precipitating circumstances among suicide decedents. The aim of this study was to determine circumstantial associations of intimate partner problem-related suicides in suicide decedents in Kentucky.

**Methods:**

All suicides that were reported to the Kentucky Violent Death Reporting System between 2005 and 2012 were eligible for this study. Multiple logistic regression was used to explore predictors (precipitating health-related problems, life stressors, and criminal/legal issues) of intimate partner problem-related suicides.

**Results:**

Of the 4,754 suicides, included in this study, approximately 17% had intimate partner problems prior to suicide. In the adjusted analysis, mental health issues, alcohol problems, history of suicides attempts, suicides precipitated by another crime, and other legal problems increased the odds of having an intimate partner-related suicide. However, having physical health problems, prior to the suicide, decreased the odds of intimate partner-related suicide.

**Conclusions:**

These results provide insight for the development of suicide interventions for individuals with intimate partner problems by targeting risk factors that are prevalent among this population. Moreover, these results may help marriage/relationship and/or family/divorce court representatives identify individuals with intimate partner problems more at risk for suicide and alleviate the influence these suicide risk factors have on individuals experiencing Intimate partner problems.

## Introduction

As the 10th leading cause of death in the United States (U.S.) in 2013, suicide is an important public health issue.^1^ Furthermore, it is imperative to understand the precipitating and historical circumstances of suicide to better identify individuals at risk for suicide to intervene more effectively. Several risk factors for suicide and suicidal behavior have been identified in prior studies. These factors include: family history of suicide, history of child maltreatment, local epidemics of suicides, media portrayal of suicides, previous suicide attempts, history of mental disorders, depression, barriers to mental health treatment, history of alcohol or substance abuse, loss (relational, social, work or financial), intimate partner problems (IPPs), physical health problems, cultural and religious beliefs that promote or glorify suicide, poverty, pregnancy hormones and easy access to lethal methods.^2-5^ Some studies also suggest that there is a difference in distribution of suicides by time of year, ^6^ time of day^7^ and criminal activities. ^8^ Moreover, risk factors for suicide vary by sex. ^9-14^

Although mental health issues are the most commonly noted precipitating circumstances in suicides and suicidal behaviors, IPPs are the second most commonly noted trigger.^15-18^ Some studies have found IPPs to be the primary circumstance among suicide decedents. ^19,20^ The Centers for Disease Control and Prevention (CDC) defines IPPs as relationship problems with current or former intimate partners. ^21^ IPPs can include divorces, separations, break-ups, arguments, jealousy, conflicts or discord. ^21^ IPPs differ from that of intimate partner violence (IPV) as IPV is described as physical, sexual or psychological harm by a current or former partner or spouse. ^22^ While IPPs can include IPV, IPPs are not limited to abuse and encompass a wide range of intimate relationship problems.

Despite the commonality of IPPs, differences between suicide decedents with precipitating IPPs and those without have not been fully studied. Most studies evaluating IPP-related suicides have focused on the differences between sex and age groups.^16,23-29^ While studies have found that older suicide decedents have lower rates of IPPs than younger age groups, ^25,27-29^ differences between males and females have been mixed. ^16,26,29^ Additionaly, IPPs and/or relationship statuses have been shown to be associated with mental health issues, physical health problems and alcohol problems; which are known risk factors for suicide. ^30-39^ While most studies have focused on demographic and health-related suicide risk factors (mental health issues, alcohol problems, and physical health problems), in persons experiencing IPPs, other important risk factors for suicide have not been studied in this population. Identifying these risk factors, such as, life stressors (financial problems, interpersonal conflicts, death of loved ones, etc.) and criminal/legal issues may provide an oppoturnity for marriage/relationship counselors to intervene and mitigate the impact these suicide risk factors have on persons experiencing IPPs.

Utilizing the Kentucky Violent Death Reporting System (KVDRS), the aim of this study was to evaluate the difference in risk factors (health-related problems, life stressors, and criminal/legal issues) for IPP-related suicides and non-IPP related suicides. Since IPPs and relationship statuses have been identified as being associated with known risk factors for suicide, it is hypothesized that individuals with mental health issues, physical health problems and alcohol problems will be more likely to have an IPP-related suicide than individuals without these issues. We also will evaluate the impact life stressors and criminal/legal problems have on IPP-related suicide.

## Methods

**Data Source**

All 4,754 suicides that were reported to the KVDRS between 2005 and 2012 were eligible for this study. The KVDRS is an active, population-based surveillance system that contains decedent demographic information and related circumstances of violent deaths (homicides, suicides, unintentional firearms deaths, and legal interventions) that occur within the state of Kentucky and is a subset of the National Violent Death Reporting System (NVDRS). ^40^ A more detailed explanation of the development and implementation of the KVDRS and the NVDRS have been previously described. ^40^ Suicides were identified by death certificates that listed International Classification of Diseases (ICD), 10th Revision codes X60-X84 and Y87.0 as the primary cause of death. ^21^ This study was approved by the University of Kentucky’s Institutional Review Board. 

**Independent Variables**

**Health-Related**

Mental health status was captured in a variety of variables in the KVDRS. This included: current depressed mood, current treatment for mental illness and ever treated for mental illness.^21^ A more detailed explanation of the variables can be found in the NVDRS coding manual. ^21^ Due to the collinearity issues with these three variables, they were combined into one variable: mental health issues. If an individual was identified as having a current depressed mood, current treatment for mental illness or was ever treated for mental illness, they were considered to have mental health issues. If an individual did not have any of these issues, prior to the suicide, they were considered to be without mental health issues. In the KVDRS coding manual, alcohol problem identified persons having a dependence or problem with alcohol. Physical health problem identified persons who had a terminal illness, debilitating disease or chronic pain that appeared to have contributed to the suicide. ^21^ Mental health issues, alcohol problem, and physical health problem were categorized dichotomously as: yes and no (reference). 

**Life Stressors**

The KVDRS includes several life stressors that are risk factors for suicide. The following life stressors were included in this study: argument over money/property/drugs; other argument/abuse/conflict; financial problem; other death of a friend or a family member; suicide of a friend or family member; and history of suicide attempts. Argument over money/property/drugs specifically relates to arguments that were related to money, property, and drugs. Other argument/abuse/conflict pertains to other types of interpersonal conflict excluding IPV, jealousy between intimate partners, and arguments over money, property, and drugs. A more detailed explanation of the variables can be found in the NVDRS coding manual. ^21^ Life Stressors were categorized dichotomously as: yes and no (reference). 

**Criminal / Legal Problems**

The KVDRS includes several variables related to criminal and/or legal problems that are associated with the risk of suicide. The following criminal/legal problems were included in this study: precipitated by another crime, recent criminal legal problem, and other legal problem. A more detailed explanation of the variables can be found in the NVDRS coding manual.^21^ Criminal/legal issues were categorized dichotomously as: yes and no (reference).

**Confounding Variables**

**Demographics**

Age at time of death was used and categorized as following: <18 to 34 years of age, 35-64 years of age, and ≥65 years of age (reference). Race was categorized as white (reference) and black. Sex was categorized as male and female (reference).

**Outcome Variable**

**IPP-Related Suicide**

IPP-related suicides are defined as problems with a current or former intimate partner that appeared to be related to the suicide.^21^ IPPs can include divorces, break-ups, arguments, jealousy, conflicts and/or discord. ^21^ IPP-related suicide was categorized dichotomously as: yes and no (reference).

**Statistical Analysis**

Descriptive statistics for the study population were calculated using frequencies and percentages. For the bivariate analysis, chi-square tests of independence, and Fisher’s exact test of independence were utilized to compare IPP-related suicides and non IPP-related suicides in terms of demographics, mental health issues, alcohol use, physical health problems and other precipitating circumstances. A multiple logistic regression model with backward elimination method was used to further explore these predictors of suicide among decedents with IPPs and decedents without IPPs. We controlled for health-related problems, life stressors, criminal/legal issues, and demographic variables, as previously described. Pearson and deviance goodness-of-fit tests were used to determine how well the model fit. After utilizing backward elimination method to inform model building, additional variables were removed manually to improve model fit statistics. A p value of <0.05 and 95% confidence intervals were used to determine statistical significance. Data was analyzed using SPSS version 21.

## Results

Table 1 displays the demographic and characteristic information of the study population. Between 2005 and 2012, 4,754 suicides were captured in the KVDRS and included in this study. Fifty-six percent of the study population was between the ages of 35 and 64 years. The majority of the population were male (81.6%) and white (95.8%). Mental health issues were the most common precipitating circumstances with approximately 35% of the population having mental health issues. The second most common precipitating circumstance was physical health problems (23%). 

**Table 1:** Suicide risk factors by intimate partner problems among suicide decedents in Kentucky: 2005-2012.

	Total Population	Intimate Partner Problem		p-value
	n (%)	Yes n (%)	No n (%)	
**Total**	4754 (100)	797 (16.8)	3957 (83.2)	
**Age Group**				<0.001
<18 to 34 years of age	1309 (27.9)	294 (22.5)	1015 (77.5)	
35-64 years of age	2639 (56.3)	471 (17.9)	2168 (82.1)	
≥65 years of age	743 (15.8)	30 (4.0)	713 (96.0)	
**Race**				0.005
White	4553 (95.8)	747 (16.4)	3806 (83.6)	
Black	157 (3.3)	41 (26.1)	116 (73.9)	
**Sex**				<0.001
Male	3879 (81.6)	685 (17.7)	3194 (82.3)	
Female	875 (18.4)	112 (12.8)	763 (87.2)	
**Mental Health Issues**				<0.001
Yes	1641 (34.5)	372 (22.7)	1269 (77.3)	
No	3113 (65.5)	425 (13.7)	2688 (86.3)	
**Alcohol Problem**				<0.001
Yes	395 (8.3)	132 (33.4)	263 (66.6)	
No	4359 (91.7)	665 (15.3)	3694 (84.7)	
**Argument over Money/Property/Drugs**				<0.001
Yes	99 (2.1)	42 (42.4)	57 (57.6)	
No	4655 (97.9)	755 (16.2)	3900 (83.8)	
**Other Argument/Abuse/Conflict**				<0.001
Yes	419 (8.8)	121 (28.9)	298 (71.1)	
No	4335 (91.2)	676 (15.6)	3659 (84.4)	
**Financial Problem**				<0.001
Yes	265 (5.6)	92 (34.7)	173 (65.3)	
No	4489 (94.4)	705 (15.7)	3784 (84.3)	
**Physical Health Problem**				0.009
Yes	1089 (22.9)	209 (19.2)	880 (80.8)	
No	3665 (77.1)	588 (16.0)	3077 (84.0)	
**Other Death of Friend or a Family Member**				0.495
Yes	171 (3.6)	28 (16.4)	143 (83.6)	
No	4583 (96.4)	769 (16.8)	3814 (83.2)	
**Suicide of a Friend or a Family Member**				0.189
Yes	65 (1.4)	14 (21.5)	51 (78.5)	
No	4689 (98.6)	783 (16.7)	3906 (83.3)	
**Precipitated by another Crime**				<0.001
Yes	47 (1.0)	20 (42.6)	27 (57.4)	
No	4707 (99.0)	777 (16.5)	3930 (83.5)	
**Recent Criminal Legal Problem**				<0.001
Yes	237 (5.0)	62 (26.2)	175 (73.8)	
No	4517 (95.0)	735 (16.3)	3782 (83.7)	
**Other Legal Problems**				<0.001
Yes	72 (1.5)	26 (36.1)	46 (63.9)	
No	4682 (98.5)	771 (16.5)	3911 (83.5)	
**History of Suicide Attempts**				<0.001
Yes	357 (7.5)	95 (26.6)	262 (73.4)	
No	4397 (92.5)	702 (16.0)	3695 (84.0)	

Table 1 also presents the bivariate analysis of suicide risk factors by IPP status. Individuals with mental health issues, alcohol problems, physical health problems, arguments over money/property/drugs, other argument/abuse/conflicts, financial problems, physical health problems, history of suicide attempts, suicides precipitated by another crime, recent criminal legal problems, and other legal problems were significantly more likely to have an IPP-related suicide than their counterparts. The remaining variables were found to be insignificant in relationship with IPPs. 

Multiple logistic regression was used to determine the association of the selected precipitating circumstances with IPPs, while controlling for confounding variables (Table 2). The final model included the following variables: mental health issues, alcohol problem, physical health problem, history of suicide attempts, precipitated by another crime, other legal problems, age, race, and sex.

**Table 2:** Multiple logistic regression of intimate partner problems by suicide risk factor of suicide decedents.

	Intimate Partner Problem			95% CI
	Yes n (%)	No n (%)	a OR	
**Mental Health Issues**				
Yes	372 (22.7)	1269 (77.3)	2.12	(1.73,2.61)
No	425 (13.7)	2688 (86.3)		
**Alcohol Problem**				
Yes	132 (33.4)	263 (66.6)	2.14	(1.68,2.71)
No	665 (15.3)	3694 (84.7)		
**Physical Health Problem**				
Yes	209 (19.2)	880 (80.8)	0.65	(0.51,0.82)
No	588 (16.0)	3077 (84.0)		
**Precipitated by another Crime**				
Yes	20 (42.6)	27 (57.4)	4.05	(2.21, 7.42)
No	777 (16.5)	3930 (83.5)		
**Other Legal Problems**				
Yes	26 (36.1)	46 (63.9)	2.11	(1.26, 3.53)
No	771 (16.5)	3911 (83.5)		
**History of Suicide Attempts**				
Yes	95 (26.6)	262 (73.4)	1.51	(1.14, 1.98)
No	702 (16.0)	3695 (84.0)		

The following confounding variables were included in the model but not in the table: age, race, and sex

As expected, individuals with mental health issues (aOR = 2.12; 95%CI = 1.73-2.61), and alcohol problems (aOR = 2.14; 95%CI = 1.68-2.71) were at significantly greater odds of having an IPP-related suicide than individuals without mental health issues and alcohol problems, respectively. Individuals with a history of suicide attempts (aOR= 1.51; 95%CI= 1.14-1.98), suicides precipitated by another crime (aOR = 4.05; 95%CI = 2.21-7.42) and other legal problems (aOR = 2.11; 95%CI = 1.26-3.53) were at greater odds of having an IPP-related suicide than those who did not have these issues.

Unexpectedly, physical health problems went from increasing the odds of IPPs in the unadjusted analysis to decreasing the odds of IPPs in the adjusted analysis. In the adjusted analysis, individuals with physical health problems (aOR = 0.65; 95%CI = (0.51-0.82) were at significantly lesser odds of having IPPs as a precipitating factor. 

## Discussion

In this study, we found that individuals with mental health issues had increased odds of having an IPP-related suicide. Previous research has shown that individuals with IPPs and/or unmarried individuals are more likely to have mental health issues than individuals without IPPs and/or married individuals.^30-34^ Therefore, it is not unexpected that mental health problems are associated with IPP-related suicides. It is also not surprising that individuals with alcohol problems were at greater odds of having an IPP-related suicide than individuals without alcohol problems, since alcoholism has been linked to higher rates IPPs (such as divorce). Individuals might abuse alcohol as a way of coping with IPPs. ^36-39^

Interestingly, in our study, we found that physical health problems were protective against IPP-related suicides. In the unadjusted analysis, individuals with physical health problems were at greater odds of having an IPP-related suicide than individuals without physical health problems. After controlling for age, race, sex, and precipitating circumstances, physical health problems became significantly protective against IPP-related suicides. 

While it is unclear why physical health problems seem to be protective of IPP-related suicides, prior research has indicated that diagnosis of physical health problems can increase marital/relationship satisfaction. A previous study, among breast cancer patients and their partners, indicated that breast cancer diagnoses brought some couples closer together.^41^ Another study also found relationship satisfaction amid physical health problems.^42^ Therefore, physical health problems may decrease the occurrence of IPP-related suicides because relationship support, empathy, and satisfaction may increase while experiencing physical health problems. Moreover, more research should be done in this area to better understand how physical health problems can have a protective relationship with IPPs. 

Suicides precipitated by a serious crime and suicide decedents with other precipitating legal problems were also associated with IPPs. Interestingly, suicides precipitated by a serious (felony) crime was one of the strongest associations with IPPs. To our knowledge, no other previous studies have evaluated suicides precipitated by another crime in relation to IPP-related suicides. More research should be done to better understand the relationship between these variables and IPP-related suicide, especially given the strong association. Since other legal issues can be defined as recent child custody disputes and/or divorce proceedings,^21^ these results need to be interpreted with caution. If an individual was identified as having other legal issues due to a conflict with a former or current intimate partner, they would automatically be identified as having an IPP-related suicide. Therefore, it is unclear if other legal issues unrelated to IPPs (child custody disputes, divorce proceedings, etc.) are significantly associated with IPP-related suicides. More research should be done to determine if other legal issues unrelated to child custody disputes and/or divorce proceedings are related to IPPs.

There are some limitations to this study. The KVDRS is limited by the information that is available at the scene of the death and the information collected by the investigator. Moreover, much of the information is obtained from interviews with friends and family members of the deceased. Therefore, the information acquired by the investigator is subject to recall bias. 

Another limitation is that this study focused on suicides that occurred only in Kentucky. Therefore, these results may not be generalizable to other populations. Future studies should consider evaluating other groups of populations or a nationally-based sample to further assess risk factors for IPP-related suicides. A future study consisting of data obtained from the NVDRS would be beneficial in studying the risk factors for IPP-related suicides.

This study is also limited by the confines that exist within the KVDRS system. While the KVDRS has an immense amount of information pertaining to violent deaths and suicides, it does not contain information about all risk and protective factors relating to suicide. Therefore, potentially confounding variables may not have been accounted for. 

The strengths of this study include the large sample size obtained from a population-based data source and data captured in the KVDRS are collected from a variety of sources. Multiple sources allow for comparisons between data sources; thereby, improving the identification of errors in reporting. Furthermore, with information being recorded from different perspectives, this dataset provides a more comprehensive analysis of suicides than datasets that only utilize one single source. 

In conclusion, this study identified several risk factors for IPP-related suicides. The results from this study may provide insight for the development of more appropriate suicide interventions for individuals with IPPs by targeting risk factors that are prevalent among this population. Marriage/relationship counselors and/or family/divorce court representatives should question individuals with IPPs about these health-related problems, life stressors, and criminal/legal issues that may influence suicidal behaviors. Additionally, they may want to develop interventions that help individuals, experiencing IPPs, manage and/or mitigate these stressors. Furthermore, the combination of IPPs, alcohol abuse and mental illness indicate that couples having conflict should be asked about suicidal ideation, attempts, depression, and substance use without judgment or consequence. Conversely, those at risk for suicide should be questioned about IPPs (i.e. suicide hotlines). 
